# Mapping urban soundscape patterns using user-generated content: A place model approach to acoustic environment perception

**DOI:** 10.1371/journal.pone.0343393

**Published:** 2026-02-24

**Authors:** Haneul Lee, Youngchul Kim

**Affiliations:** 1 KAIST Urban Design Lab, KAIST Smart City Research Center, Department of Civil and Environmental Engineering, Korea Advanced Institute of Science and Technology, Daejeon, Republic of Korea; 2 KAIST Urban Design Lab, KAIST Smart City Research Center, Department of Civil and Environmental Engineering, Korea Advanced Institute of Science and Technology, Daejeon, Republic of Korea; Shenyang Jianzhu University, CHINA

## Abstract

Urban acoustic environments significantly influence quality of life, prompting a paradigm shift from noise reduction to soundscape design that considers human perception. This study investigates urban soundscape perceptions using social media data to understand how acoustic environments reflect urban spatial characteristics. We collected Korean-language Twitter texts from 156 points of interest in Seoul’s central and southeastern neighborhoods from October to November 2020. The collected texts were preprocessed through text cleaning and morphological analysis, then classified using an urban soundscape taxonomy based on Canter’s place model that integrates physical setting, activity, and meaning dimensions. The results revealed that activity-related soundscapes (79.4%) dominated all target areas, and strong negative correlations between indoor, mechanical, and behavioral soundscapes suggest a spatial separation of acoustic zones by urban function. Especially, historical central districts (22.0%) showed a higher proportion of meaning-related soundscapes compared to modern commercial districts (13.4%). K-means clustering further identified three soundscape types: infrastructure-dominated, socially vibrant, and commercial indoor-focused zones. By combining social media analysis with spatial patterns, the study provides a scalable tool for urban planners to understand how citizens experience urban soundscapes, offering data-driven insights for contextualized urban design.

## 1. Introduction

Noise is commonly defined as unwanted sound that can cause physiological and psychological disturbances to humans [1]. Long-term exposure to urban noise is related to various health issues, including stress and cardiovascular disease [2–4]. Traditionally, urban acoustic environment management has focused on reducing sound pressure levels (dB) [5,6]. However, this approach is limited as it does not consider the quality of the acoustic environment, where a low sound pressure level does not always correlate with human well-being [7].

Recognizing these limitations, the EU has sought solutions based on soundscapes [8]. A soundscape is the perceived acoustic environment in context by an individual, group, or society [9]. Unlike traditional noise management that focuses solely on quantitative reduction, the soundscape approach emphasizes qualitative acoustic environments that distinguish between wanted and unwanted sounds, utilizing preferred sounds to stimulate human senses [5,8]. At the urban scale, soundscape characteristics are influenced by individual attributes, sociocultural factors, physical environment, and geographic features [8,11–15], making them indicators of urban identity. Therefore, analyzing urban soundscapes is essential for creating pleasant acoustic environments through urban planning [8].

R. Murray Schafer [10] established an initial classification derived from historical and literary documents and classified soundscape elements into six functional categories: natural sounds, human sounds, society, mechanical sounds, indicators, quiet, and silence. This led to empirical standardization for managing the acoustic environment. Brown et al. [16] proposed a framework to ensure research comparability, categorizing sources by their origin (e.g., sounds generated by human activity) rather than their perceived quality. Aiello et al. [17] used social media data to derive taxonomies based on public perception (e.g., transport, music, and indoors), while Salamon et al. [18] developed an application-oriented taxonomy for machine learning, focusing on urban sound issues identified from noise complaints (e.g., jackhammer and siren). Recent studies have moved from classifying sound sources to evaluating perceptual experience with the two-dimensional model of pleasantness and eventfulness [19,20]. Several taxonomies have emerged including functional classifications distinguishing disruptive and supportive sounds [21], cognitive process models progressing from sound classification to judgment [22], and activity-based frameworks measuring soundscape appropriateness for specific contexts such as working and relaxing [23].

However, these studies reveal limitations in covering various urban characteristics. Findings from context-specific studies during exceptional circumstances demonstrated narrow applicability to diverse urban settings [23,24]. Moreover, current taxonomies inadequately capture how soundscape perception varies according to social context, personal activity, and individual expectations [16,22]. These suggest the need for more comprehensive taxonomies that integrate perceptual features with diverse urban contexts including place type and human activity. This approach supports the concept of soundscape frameworks that emphasize the relationship between human experience and environmental context. According to Canter’s place model [25], the sense of place can coordinate a relationship between humans and the built environment and can create an appropriate function of a place. This model explains that a sense of place can also provide people with interest, a sense of belonging, and identity in urban space [25].

Common soundscape measurement methods formalized in ISO/TS 12913–2:2018 standard include on-site soundwalks, laboratory listening experiments, and narrative interviews [8,26–32]. Questionnaire-based protocols dominate the field, used in over 94% of recent studies through soundwalks or surveys [33]. However, these measurements face significant constraints including the practical difficulty of coordinating large groups and spatial and temporal restrictions. Aletta & Torresin [33] revealed that the standard’s predefined attributes often prove unsuitable for diverse contexts (e.g., indoor environments). To overcome these challenges, researchers have increasingly adopted social media and crowdsourcing approaches.

Social media platforms provide valuable sources for studying urban perceptions in multiple sensory dimensions. Dunkel [34] used crowdsourced photos to capture urban perceptions, Quercia et al. [35] mapped smellscapes across seven European cities using social media data, and Redi et al. [36] classified ambiance terms from Flickr photo tags. Soundscape research has particularly benefited from crowdsourcing methods. D’Hondt et al. [37] examined participatory noise mapping, Aiello et al. [17] linked sound-related tags to street-level data for sonic mapping, and Gasco et al. [38] analyzed citizen reactions to urban noise.

Recent advances have enhanced these approaches. Zhao et al. [39] combined street view imagery with machine learning to predict soundscapes, offering cost-effective alternatives to conventional acoustic monitoring. Gasco et al. [40] showed that integrating social media with traditional noise mapping improves health predictions by considering sound types. Despite progress, challenges remain including limited geospatial information and representation biases [41,42], making comprehensive spatial analysis of large urban soundscape difficult.

This study aims to address the limitations of previous research: 1) the insufficiency of current taxonomies to integrate the multidimensional urban context, such as place type and human activity, and 2) the spatial and temporal constraints of conventional research methods. To overcome these challenges, this study first applied Canter’s place model to develop a comprehensive soundscape taxonomy that integrates the physical setting, behavioral aspects, and semantic dimensions. Using this taxonomy, we collected and extracted keywords from text-based social media data with a georeferencing approach based on points of interest (POIs) for Seoul’s central and southeastern areas. We then mapped soundscapes by region using geographic information systems (GIS) and analyzed their characteristics. Finally, to identify the patterns in urban soundscape perception, we conducted k-means clustering analysis. By establishing a spatial typology applicable to urban planning and management, this study provides a scalable framework for designing contextualized acoustic environments.

## 2. Materials and methods

**Fig 1:**
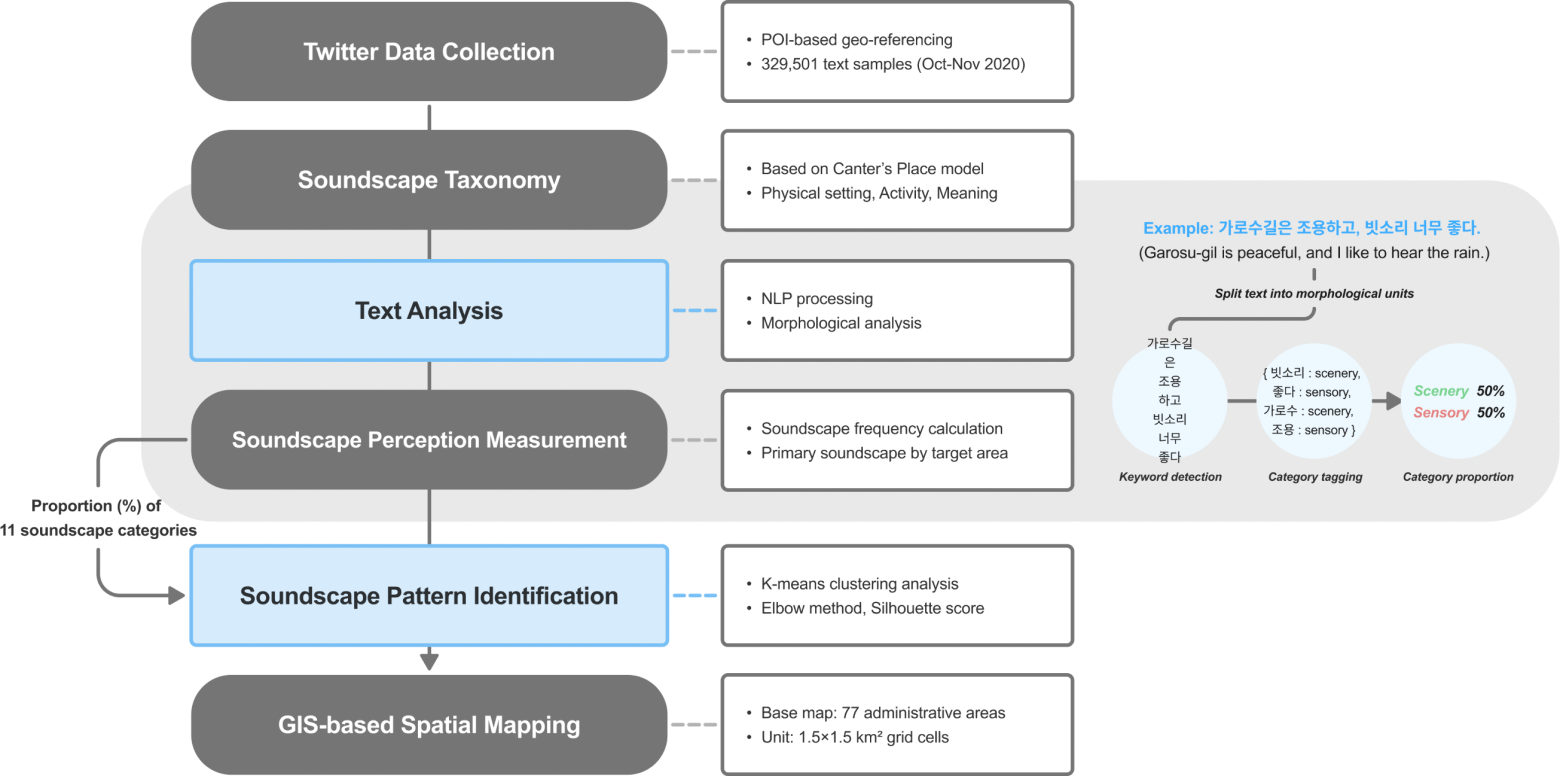
Research process.

This study follows the research process shown in Fig 1 for data collection, text analysis for the measurement of urban soundscape perception, and pattern identification of urban soundscape perception.

### 2.1. Site selection

**Fig 2:**
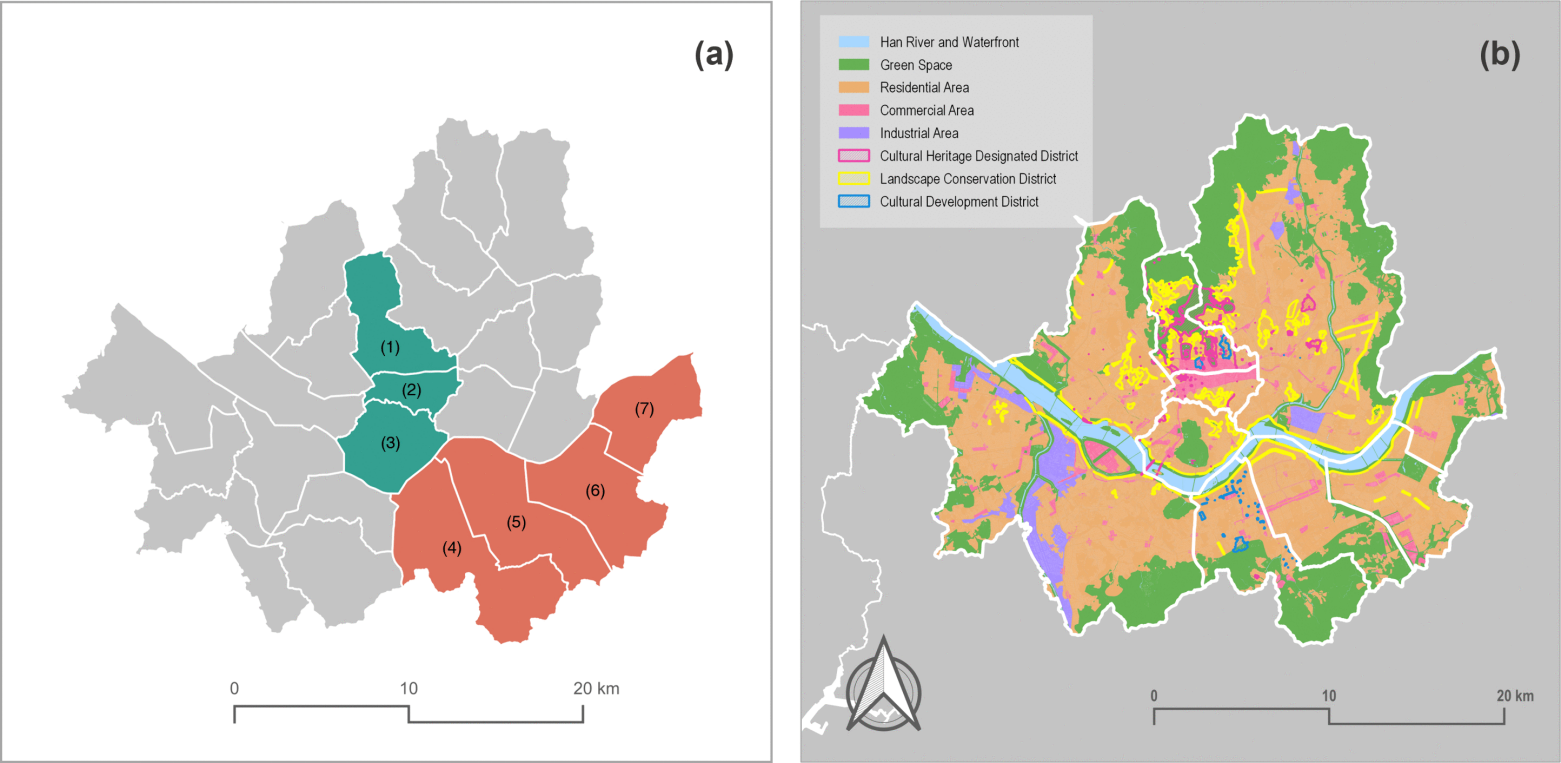
Target sites of this study. **(a)** Central and Southeastern districts in Seoul. (1) Jongro-gu; (2) Jung-gu; (3) Yongsan-gu; (4) Seocho-gu; (5) Gangnam-gu; (6) Songpa-gu; (7) Gangdong-gu. The central area includes areas 1-3, and the southeastern area includes areas 4-7; **(b)** Spatial characteristics of Seoul, including land uses and zoning areas; Source: Ministry of the Interior and Safety (http://www.juso.go.kr/externalLink/goUrl.do?menuId=DT05) and Seoul Metropolitan Government (https://data.seoul.go.kr/dataList/OA-21127/S/1/datasetView.do, https://data.seoul.go.kr/dataList/OA-21133/S/1/datasetView.do, https://data.seoul.go.kr/dataList/OA-21136/S/1/datasetView.do, https://data.seoul.go.kr/dataList/OA-21137/S/1/datasetView.do). These public works are used according to Korea Open Government License (KOGL).

We selected Seoul as the spatial scope of this study due to its active social media usage and the possibility of obtaining a sufficient data sample [43,44]. Seoul is the capital of South Korea and has an area of 605.25 ${km}^{\text{2}}$, a population of approximately 10 million, and 25 districts. The central area and the southeast area were targeted for the soundscape analysis (Fig 2). These two areas are located in the northern and southern parts of Seoul, and have different urban development patterns. The central area includes three neighborhoods: Jongro-gu, Jung-gu, and Yongsan-gu. It is a cultural, economic, and livelihood core with a rich history [45]. Also, this area is a mix of historical and cultural heritage sites such as Gyeongbokgung Palace and Deoksugung Palace, natural landscapes including Cheonggyecheon Stream and Namsan Mountain, and a downtown commercial district and low-rise residential areas. In terms of land use, it is mostly dominated by commercial and business functions, with a high concentration of tourist attractions and cultural facilities. In contrast, the southeastern area, which consists of four neighborhoods, Seocho-gu, Gangnam-gu, Songpa-gu, and Gangdong-gu, is an established residential and commercial district developed since the 1970s. It is characterized by a planned grid network of roads and high-rise apartment complexes and has emerged as a new economic zone in Seoul with strengthened business and commercial functions [45]. It is mainly composed of large-scale residential complexes and modern commercial districts, with business districts such as Teheran-ro and Garosu-gil, and public facilities including Jamsil Sports Complex and Hangang Park. Thus, by comparing two neighborhoods with different urban development backgrounds and spatial structures, it can effectively examine the relationship between urban context and soundscape perception.

### 2.2. Data collection

The data collection methods used in previous studies, such as onsite surveys and sound measurements, have limitations when considering large urban areas [8,15,26–33]. In contrast, social media data offer an alternative with fewer time and space constraints. Many samples can be captured to provide information on collective experiences in the built environment [17,34–38,46,47]. Furthermore, the use of social media data can make it easier to verify human perceptions of urban space and to comprehensively analyze these perceptions with other spatial data [17,35]. For example, Jang & Kim [46] demonstrated that the perception of a place can reflect the place’s identity using Instagram hashtag data and proposed a GIS-based cognitive map. This study expanded their approaches to explore the spatial characteristics of urban soundscapes using user-generated geographic information based on POIs.

Tweepy, a Python API library, was used to create an automated crawler for collecting Twitter data [48]. Twitter is a real-time text-based social media platform that is optimal for analyzing users’ immediate interest and reactions to specific issues [49–51]. Due to enhanced privacy policies, directly collecting location-tagged data was not feasible. To overcome this, we collected Korean-language posts based on a predefined set of 156 POIs within Seoul, ensuring spatial representativity. These POIs were selected from public data provided by the Seoul Metropolitan Government and Naver Map, one of the largest search engines and map services in Korea. Selected POIs include a mix of major tourist attractions, cultural facilities, and public spaces (e.g., Gyeongbokgung Palace, Gangnam Station, Jamsil Sports Complex, and Namsan Park). The full list of POIs is provided in Table A in S1 Appendix. We retrieved posts containing POIs in Tweepy queries, and if a location was missing a POI, we used the name of the administrative area in the query. Spatial differences on POI distribution between central and southeast neighborhoods were confirmed using Chi-square tests and Mann-Whitney U tests. While we acknowledge that not all georeferenced posts reflect direct acoustic experiences, we assume that texts associated with specific POIs are likely to capture in-situ perceptions related to those locations.

In total, 329,501 posts were collected from October to November 2020. It is important to note that this data collection period coincided with the COVID-19 pandemic, a unique temporal context may have influenced urban activity and soundscapes. This limitation will be further addressed in the discussion. The distribution of the acquired text data by neighborhood are shown in Table 1.

**Table 1:** Acquired texts by district.

Period	2020. 10. ~ 2020. 11. (60 days)		
Neighborhood	District	Number of posts	Percentage*
**Central**	Yongsan-gu	46,685	14.17%
	Jongro-gu	98,757	29.97%
	Jung-gu	46,483	14.11%
**Southeastern**	Gangnam-gu	80,381	24.39%
	Gangdong-gu	7,277	2.21%
	Seocho-gu	31,478	9.55%
	Songpa-gu	18,440	5.60%
**Total number of posts**		329,501	100.00%

*In Table 1, percentage refers to the number of posts from each district compared to the total number of posts.

### 2.3. Urban soundscape taxonomy

This study developed an urban soundscape taxonomy based on David Canter’s place model [25] to comprehensively analyze soundscape perception from social media data. The taxonomy categorizes sound-related keywords from collected text data into three dimensions: physical setting, activity, and meaning (image). To construct the taxonomy, we first reviewed previous studies on soundscape and urban perception to establish 11 main categories and 28 sub-categories corresponding to the three dimensions of place model [10,16–18,36]. We then defined Korean keywords relevant to auditory experiences based on Standard Korean Language Dictionary and KNU Korean Sentiment Lexicon [52] and assigned them to one of sub-categories.

**Fig 3:**
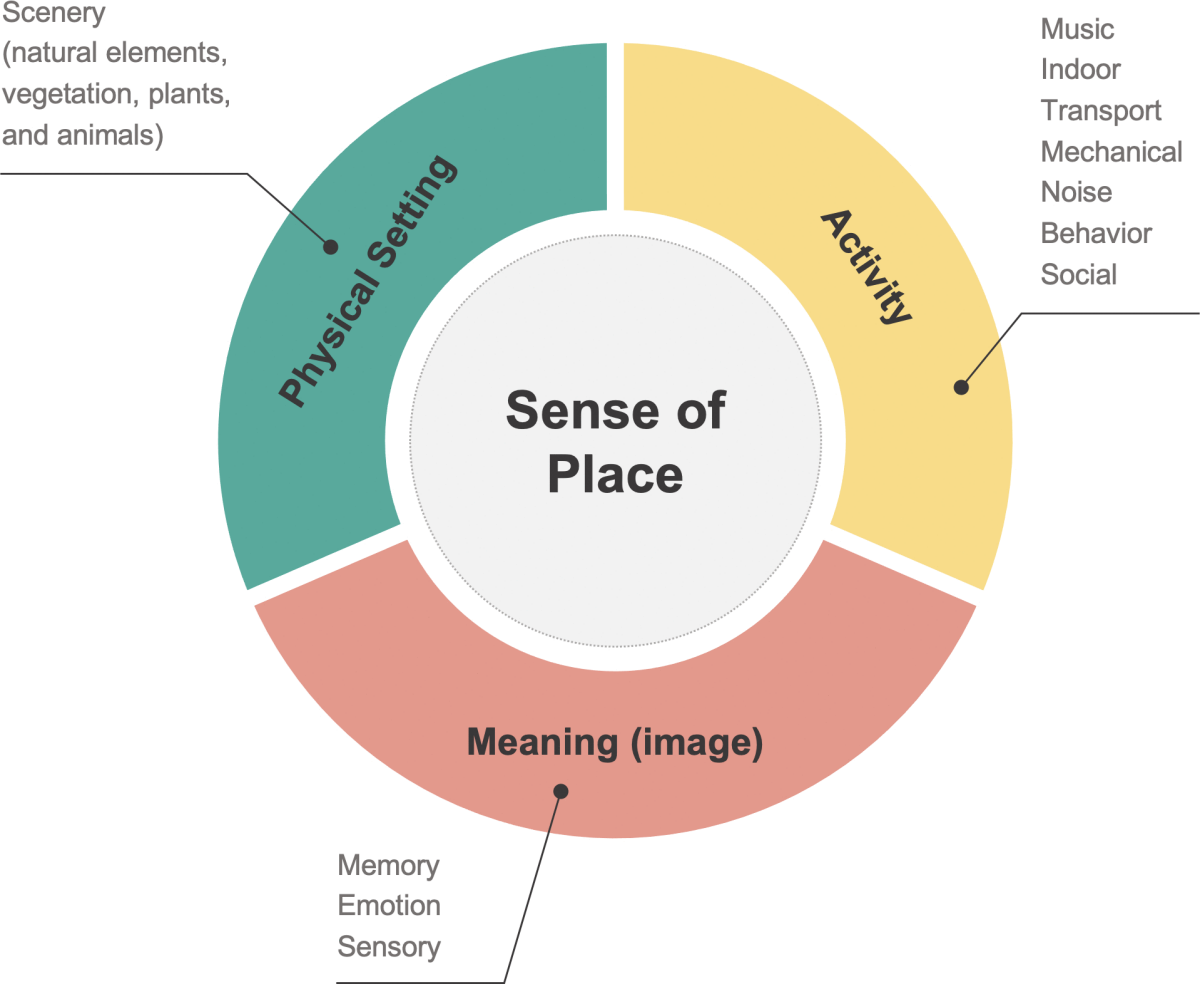
Urban soundscape taxonomy adapted from the place model. This consists of a sense of place, including physical setting, activity, and meaning (image).

Fig 3 shows the proposed soundscape taxonomy, including sound sources and semantic categories. The ‘Physical setting’ dimension includes keywords related to natural elements, such as wind and birdsong, under the ‘scenery’ main category. The ‘Activity’ dimension, which explains human activity, contains seven main categories like ‘music’, ‘indoors’, ‘transport’, ‘mechanical’, ‘unclassified noise’, ‘behavior’, and ‘social’. Lastly, the ‘Meaning’ dimension captures subjective experiences, with three main categories including ‘memory’, ‘emotion’, and ‘sensory’. This expanded classification system captures physical, behavioral, and semantic characteristics of urban soundscapes. The full list of sub-categories and keywords is provided in Table B in S1 Appendix.

### 2.4. Measuring urban soundscape perception on social media

Considering the characteristics of Korean with its complex morphemes, it is necessary to separate words into analytic forms for measuring soundscape keywords with the built soundscape taxonomy. Therefore, to refine the raw data and obtain keywords, a systematic text preprocessing was implemented. The main steps included: 1) removing identical posts (e.g., promotional content) to ensure the independence of each data point; 2) cleaning non-essential text elements such as non-Korean languages, URLs, hashtags, emoticons, special characters, and usernames; 3) performing morphological analysis on the cleaned Korean text using the Python library KoNLPy [53], primarily extracting nouns and adjectives which are most likely to contain descriptive information about soundscape perception; and 4) Filtering out a defined Korean grammatical stop words (e.g., particles) that provide little semantic value to improve the quality of the keyword analysis. After this preprocessing, the cleaned text data was used for the soundscape categorization. The soundscape taxonomy was converted to the form of a dictionary, and the refined words from the texts were matched with the categories of the soundscape dictionary. Based on this, the frequency of soundscape keywords in each neighborhood was calculated. To examine the distribution of soundscape categories by area, the frequency calculated for each of the 11 categories within the taxonomy was converted into proportions:


$$P_{i,c}=\frac{\sum_{k\in K_{C}}F_{i,k}}{\sum_{k\in K_{all}}F_{i,k}}\times \text{1}\text{0}\text{0}\;(\%)$$ (1)

This formula calculates the proportion (Pof a specific soundscape category (C) in a given area (i) by summing the frequencies (F) of all keywords within that category and dividing by the total frequency of all keywords in the area. The dominant soundscape for each area was determined based on the highest proportion among 11 soundscape categories:


$$D_{i}={argmax}_{k}\left(P_{i,C}\right)$$ (2)

This formula identifies the dominant soundscape (D) for an area (i) by finding the soundscape category (kthat has the maximum proportion (P) among the 11 categories. When the maximum values were identical, it was defined that two or more primary soundscapes are present. To quantitatively support the descriptive findings, several statistical analyses were conducted using SciPy package [54]. Spearman rank correlation (ρ) examined relationships between POI density and soundscape frequency, and Pearson correlation explored relationships among soundscape categories. The distribution of soundscape categories between central and southeast neighborhoods was validated using Mann-Whitney U tests.

### 2.5. Patterns in urban soundscape perception

The complex and multidimensional features of soundscape data make it difficult to identify overall urban patterns with individual neighborhood level analysis. Therefore, we performed k-means clustering to explore structural patterns in urban soundscape perception of the target sites. This unsupervised method groups data into a predetermined number of clusters, with each point belonging to the cluster whose centroid is most proximate.

The input for the clustering analysis was a dataset where each of the 77 administrative areas (i.e., 77 grid cells) was represented by the proportional values (%) of the 11 soundscape categories. To effectively visualize and interpret structural patterns within this data, a dimensionality reduction step was necessary. For this purpose, we adopted the Uniform Manifold Approximation and Projection (UMAP) algorithm [55]. UMAP is a technique that preserves the essential structure of the original high-dimensional data while converting it into a lower-dimensional space, in our case, two dimensions. This process makes the data more suitable for clustering analysis and interpretation.

**Fig 4:**
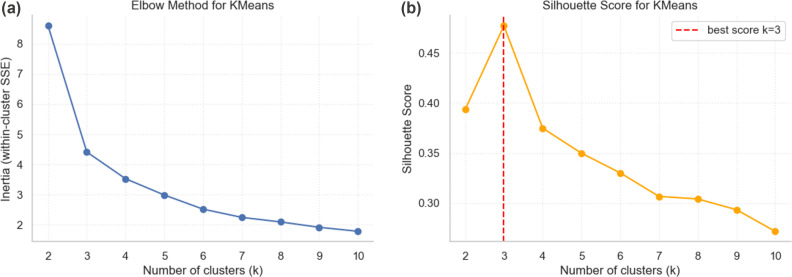
The result of Elbow method and silhouette measurement. **(a)** Elbow method shows that the rate of decrease in WCSS (inertia) slows significantly after 𝐊=3; **(b)** Silhouette score shows the highest score at 𝐊=3.

Following dimensionality reduction, we performed k-means clustering on the 2-dimensional UMAP output to group target areas with similar soundscape characteristics using Scikit-learn package [56]. Identifying the optimal cluster number (K) is important in k-means. We applied the Elbow method first, plotting the within-cluster sum of squares (WCSS) across different K values. We then performed silhouette analysis to verify clustering quality. Silhouette scores range from −1–1, measuring how fit each point is to its assigned cluster compared to other points. A higher score indicates an improved cluster allocation. Fig 4 showed that three clusters (K=3) were the optimal number, as this is the elbow point where increasing K no longer yields a significant decrease in WCSS. Also, the average silhouette score for the K=3 model was 0.36, suggesting that the clusters are reasonably distinct.

Based on these results, we finalized the model with three clusters. Using Statsmodels package [57], one-way ANOVA and post-hoc test (Tukey’s HSD) validated cluster differences for each soundscape category, with p < 0.05 considered statistically significant. This approach allowed us to compress the complex soundscape characteristics of target sites into three understandable patterns, objectively identifying correlations between different soundscape types. This classification with similar soundscape characteristics helps reveal spatial typologies that can be used for urban planning and management.

### 2.6. Spatial mapping

To visualize the geographical context and patterns of soundscape perception, the results were presented as a spatial map. The spatial analysis and mapping were conducted using QGIS, an open-source Geographic Information System (GIS) software. The base map, which contains the administrative boundaries of the 77 areas in the target sites, was created using a shapefile obtained from the open data portal of the National Geographic Information Institute (NGII) of South Korea. The base map was converted into 1.5 × 1.5 ${km}^{\text{2}}\;$grid cells to show the primary results of the target sites by covering each area. Next, the soundscape metrics calculated for each area, such as the soundscape patterns and the dominant soundscape with the highest proportion across all categories, were spatially joined to grid cells. This process enabled the creation of visualized maps that effectively show the spatial distribution and characteristics of urban soundscapes in the target sites.

## 3. Results

### 3.1. Descriptive statistics

**Fig 5:**
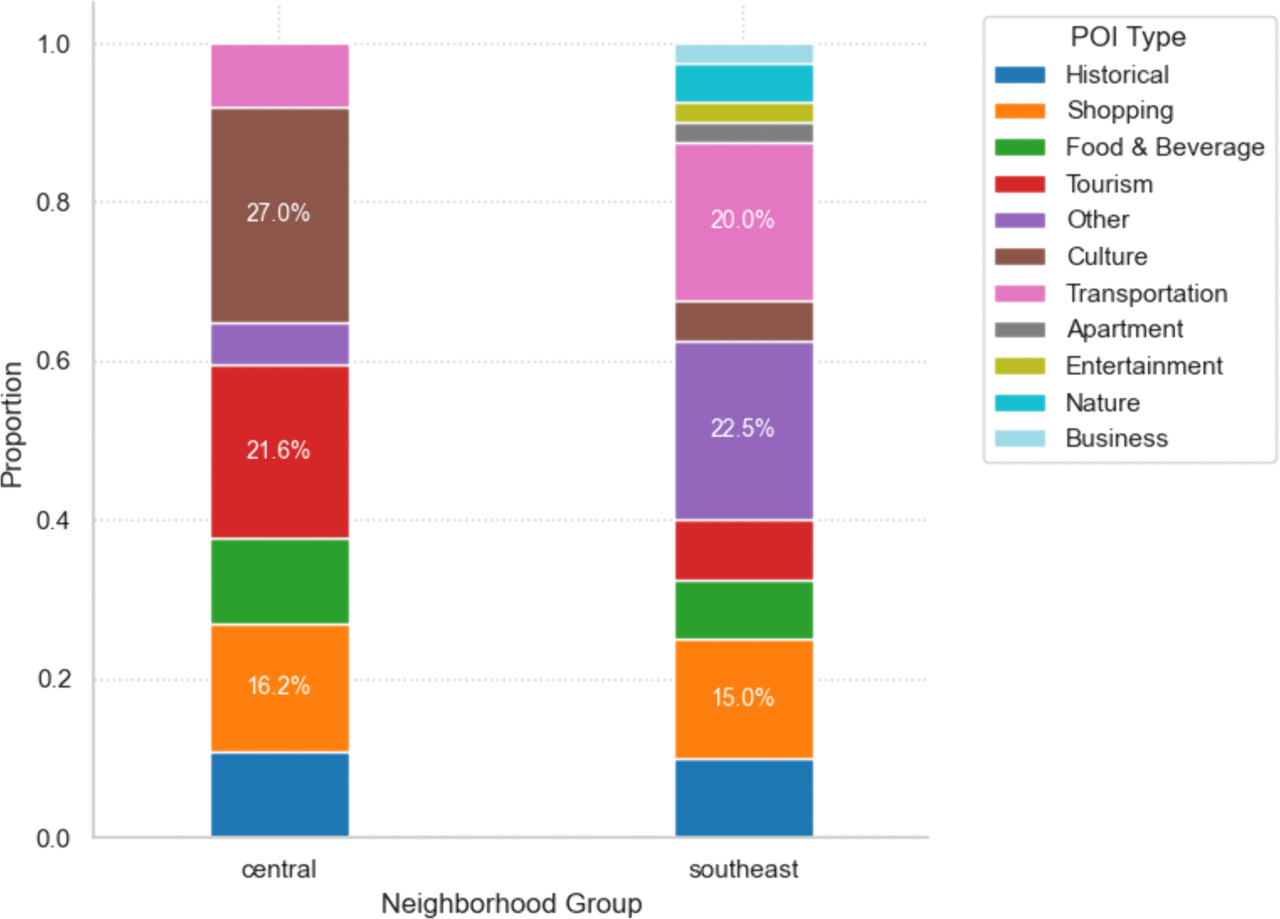
POIs type distribution by neighborhood group. This illustrates the difference in POI types between the central area and the southeastern area. Particularly, the central area, which has a longer history compared to the southeastern area, was found to have more attraction-related POIs (i.e., historical, tourism, culture, shopping, and entertainment) (Mann-Whitney test, p < 0.001).

Table 2 shows the descriptive statistics of the target areas. A total of 143,100 soundscape frequencies of target sites were extracted from the original posts. To enable valid comparisons between neighborhoods with varying levels of social media activity, all absolute frequencies of soundscape keywords were normalized into proportional data (%). While Jongro-gu had the highest absolute frequency of total texts (98,757), the frequency of soundscape keywords reveals a different pattern. When calculated as the proportion of soundscape keywords to total posts, Gangnam-gu showed the highest percentage (68.3%), followed by Seocho-gu (42.8%) and Jung-gu (38.1%). Gangdong-gu exhibited the lowest proportion (18.7%), indicating that soundscape documentation varies significantly by districts beyond the simple number of posts. The central neighborhood is older than the southeast neighborhood and has more attraction-related POIs, such as historical, tourism, culture, shopping, and entertainment types (Mann-Whitney U test, p < 0.001) (Fig 5). A significant positive relationship was found between attraction-related POI density and soundscape frequency (Spearman ρ = 0.43, p < 0.001), suggesting that the presence of numerous attractions are strongly related to acoustic experience.

**Table 2:** Descriptive statistics of the target areas.

Neighborhood	District	Soundscape frequency	Percentage^a^	Total number of POIs	Primary category of POIs
Central	Yongsan-gu	16,421	35.17%	27	Culture
	Jongro-gu	33,314	33.73%	40	Culture
	Jung-gu	17,690	38.06%	29	Culture
Southeastern	Gangnam-gu	54,938	68.35%	15	Transportation
	Gangdong-gu	1,363	18.73%	13	Other^b^
	Seocho-gu	13,484	42.84%	13	Tourism
	Songpa-gu	5,890	31.94%	19	Shopping
Total		143,100			

The types of POIs are classified as follows: Tourism, Culture, Shopping, Entertainment, Transportation, Food & Beverage, Nature, Business, Apartment, Accommodation, Historical, and Other.

a In Table 2, percentage refers to the frequency of the soundscape keywords in the number of posts by each district.

b This type includes areas or specific places that are not covered by the primary POI categories.

As shown in Table 3, the presence of a high-ranking soundscape frequency is associated with human activities (e.g., restaurants, cafés, and events). This pattern shows that social media users actively document and share their acoustic experiences in spaces where social interactions occur, reflecting the relationship between human activities and soundscape perception. The prominence of informative text and personal impressions about these spaces suggests that soundscapes play a significant role in how people experience and perceive urban places.

**Table 3:** Soundscape keyword ranking (Top 20) in collected texts from social media.

Sense of place	Keyword	Soundscape category	Frequency	Rank
Physical setting	vegetation, plants, and animals	Scenery	1,485	12
	natural elements	Scenery	705	16
Activity	live	Music	374	18
	restaurant	Indoor	41,154	1
	cafe	Indoor	33,815	2
	store	Indoor	1,179	15
	car, motorcycle, bus, truck	Transport	3,540	8
	subway	Transport	2,060	9
	train	Transport	178	20
	construction	Mechanical	9,779	5
	event	Behavior	14,448	3
	adult	Behavior	9,067	6
	children	Behavior	5,016	7
	voice	Behavior	2,009	11
	movement	Behavior	1,443	13
Meaning (image)	information	Sensory	12,293	4
	ambiance	Sensory	635	17
	positive	Emotion	2,018	10
	negative	Emotion	1,308	14
	neutral	Emotion	187	19

### 3.2. Urban soundscape perception

**Fig 6:**
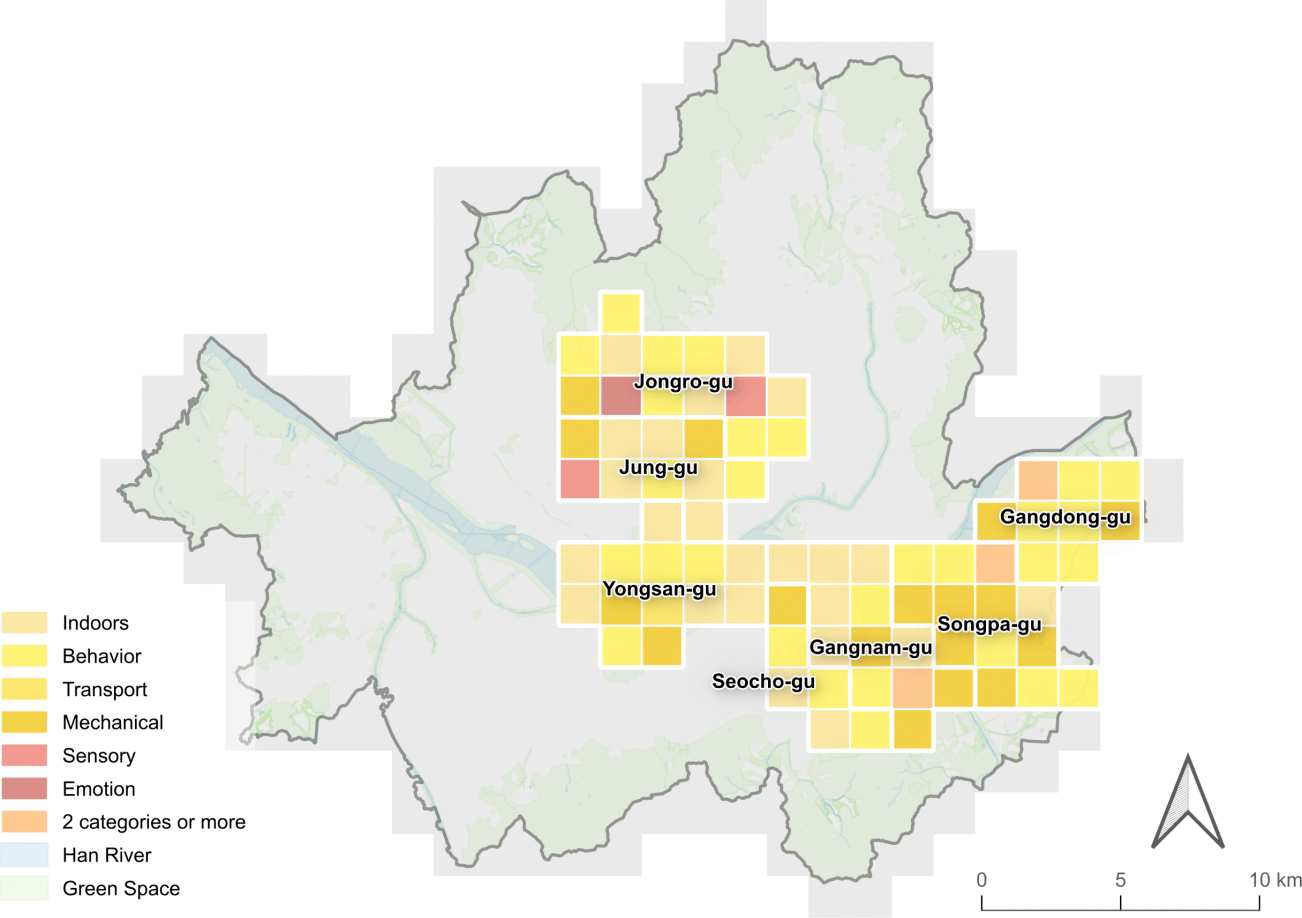
Dominant urban soundscapes by district. This map depicts the target area in 1.5 × 1.5 ${𝐤𝐦}^{\text{2}}\;$ grid cell units to identify the spatial distribution of soundscape types. It shows the primary soundscape categories for 77 sub-regions, specifically the category with the highest proportion. Where categories have the same proportion, they are represented as two or more; Source: Ministry of the Interior and Safety (http://www.juso.go.kr/externalLink/goUrl.do?menuId=DT05) and Seoul Metropolitan Government (https://data.seoul.go.kr/dataList/OA-21136/S/1/datasetView.do). These public works are used according to Korea Open Government License (KOGL).

**Fig 7:**
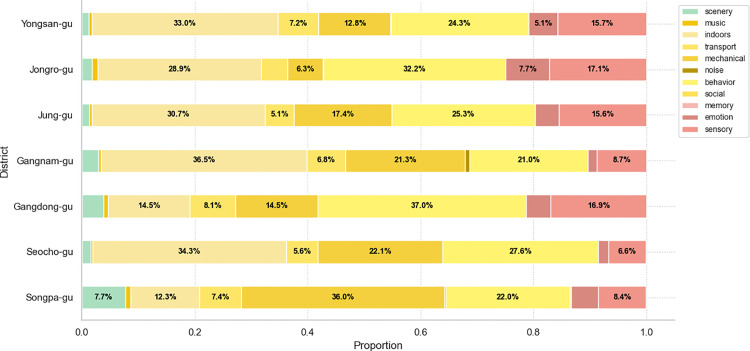
Proportion of urban soundscapes by district. It displays the proportion and composition of soundscape categories in each district. Activity-related soundscapes are dominant across all districts.

The classification of soundscape types revealed the following distinct spatial patterns in Seoul’s districts: 1) indoor environments in Jung-gu (30.7%), Yongsan-gu (33.0%), Seocho-gu (34.3%) and Gangnam-gu (36.5%), 2) behavioral activities in Jongro-gu (32.2%) and Gangdong-gu (37.0%), and 3) mechanical activities in Songpa-gu (36.0%) (Figs 6 and 7). Among the 77 analyzed grid cells from target sites, behavior-related sounds were most frequently dominant (n = 29), followed by indoor sounds (n = 24) and mechanical sounds (n = 17). Activity-related soundscapes (i.e., music, indoors, noise, transport, mechanical, behavior, and social) comprised an average of 79.4% across all neighborhoods (Mann-Whitney U test, p < 0.001), with Seocho-gu showing the highest proportion (89.9%) and Jongro-gu the lowest (73.4%). This pattern shows that social media users primarily share acoustic experiences related to human activities rather than ambient environmental sounds. The consistency of this pattern across all districts suggests that people tend to capture acoustic experiences of their daily urban interactions.

#### 3.2.1. Physical setting.

**Fig 8:**
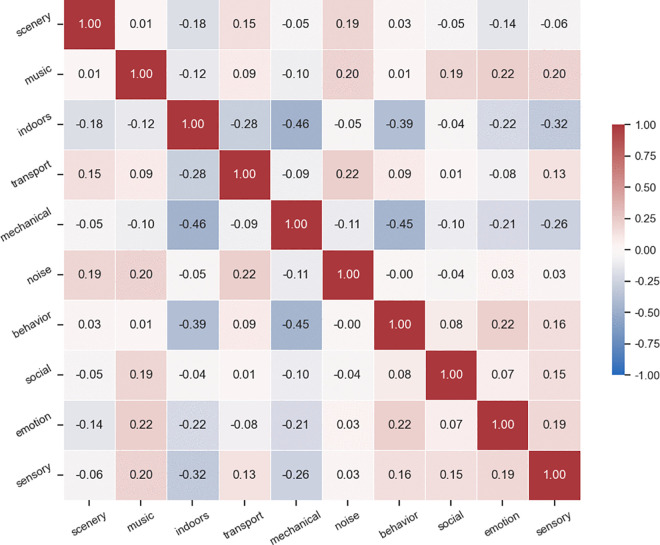
Pearson correlation matrix of soundscape categories. This shows correlations between soundscape categories. Especially, indoor-mechanical (r = −0.46), mechanical-behavior (r = −0.45), and indoor-behavior (r = −0.39) soundscapes exhibit relatively strong negative correlations. The Memory category was excluded from the analysis because no keywords were found in all regions.

Physical setting soundscapes showed limited presence across all target areas (Fig 7). The only notable scenery-related content was found in Songpa-gu (7.7%) and can be attributed to weather conditions, such as wind. Correlation analysis showed that physical soundscape categories (i.e., scenery) had weak relationships with other soundscape types (|r| < 0.2), suggesting these natural and ambient sounds exist independently from activity-based urban sounds (Fig 8). This reveals a fundamental characteristic of social media-based soundscape research, where natural acoustic elements are less prevalent in digital content than human-generated sounds.

The lack of natural sound elements, even in areas with parks as POIs, suggests that social media users prioritize socially engaged acoustic experiences compared to environmental soundscapes. Therefore, traditional soundscape assessment methods are still necessary for a comprehensive evaluation of the acoustic environment. However, this also provides an important insight since the soundscapes people share and discuss reflect the acoustic elements that significantly impact their urban experience and sense of place. Additionally, the scarcity of natural soundscapes in social media data indicates potential opportunities for urban acoustic design. Enhancing or introducing natural sounds in such areas could increase the biodiversity of acoustic experiences, changing public perception on social media representation.

#### 3.2.2. Activity.

Activity soundscapes showed high proportions in both central and southeastern neighborhoods (76.5% and 82.0%), confirming that human-generated acoustic experiences are essential to urban soundscape perception based on social media data (Figs 6 and 7). Correlation analysis revealed important spatial relationships between different activity soundscapes (Fig 8). Strong negative correlations were found between indoor and mechanical soundscapes (r = −0.46), mechanical and behavior soundscapes (r = −0.45), and indoor and behavior soundscapes (r = −0.39). These negative correlations indicate that these soundscape types tend to occur in spatially distinct areas rather than overlapping, implying that different urban functions create separate acoustic zones. This finding reinforces previous research and expands understanding of how various human activities produce unique soundscapes that influence urban residents [10,16–18,36].

Behavioral soundscapes concentrated in cultural, leisure, and tourism areas, such as galleries, palaces, traditional markets, cultural complexes, and landmarks, particularly in districts with higher densities of cultural POIs (Chi-square test, p = 0.039). The negative correlation with mechanical sounds (r = −0.45) suggests that areas with high social activity are spatially separated from infrastructure-dominated zones. Keywords associated with events, voices, adults, children, and movement suggest that these spaces are perceived as vibrant acoustic environments, where human activity plays a key role in shaping the soundscape. Thus, cultural preservation should consider acoustic heritage with visual and architectural elements.

Indoor soundscapes were prevalent in commercial districts, specifically in areas with retail facilities and street-level businesses, such as cafés, restaurants, stores, and shopping malls. The strong negative correlation with mechanical sounds (r = −0.46) indicates that commercial indoor spaces and infrastructure zones form distinct acoustic areas within the city. The prevalence of indoor soundscapes reflects how social media users perceive commercial spaces that facilitate social interaction through acoustic control. Meanwhile, the negative relationship with behavioral soundscapes (r = −0.39) demonstrates that indoor commercial activities and outdoor social behaviors cover different urban spaces. This suggests that urban commercial areas need to focus on indoor environments to support the social activities that drive their vitality.

Mechanical soundscapes appeared near infrastructure facilities, showing inverse relationships with both indoor commercial and outdoor behavioral sounds. Keywords such as ‘construction,’ ‘vehicle,’ ‘train,’ ‘subway,’ and ‘airplane’ show that transportation and infrastructure sounds lead public perception about outdoor soundscapes. Many areas with dominant mechanical soundscapes were residential, which implies that infrastructure noise significantly impacts the quality of life in those areas. These spatial separations of acoustic experience provide the foundation for understanding the three distinct soundscape patterns identified from urban landscape of target sites (see the Urban soundscape patterns by clustering analysis section).

#### 3.2.3. Meaning (Image).

Meaning-related soundscapes presented a spatial concentration in Jongro-gu and Jung-gu, which are areas of significant historical and cultural heritage (Fig 6). Statistical analysis confirmed that central neighborhoods (22.0%) had significantly higher proportions of meaning-related soundscapes compared to southeast areas (13.4%) (Mann-Whitney U test, p = 0.004). Specifically, emotion-related soundscapes were nearly twice as common in central areas (5.8%) compared to southeast areas (3.3%), while sensory-related soundscapes also showed higher prevalence in central districts (16.2% vs 10.2%). This demonstrates how place identity influences the emotional and sensory aspects of acoustic experiences.

Remarkably, while sensory soundscapes showed weak negative correlations with indoor (r = −0.32) and mechanical (r = −0.26) sounds, they demonstrated positive associations with behavioral soundscapes (r = 0.16). This pattern suggests that sensory experiences are more likely to be documented in socially active spaces rather than commercial indoor or infrastructure-dominated areas. The contrast between the central and southeastern neighborhoods revealed statistically significant difference in meaning-related soundscape expressions. Southeastern areas have more developed commercial facilities but generate fewer of these soundscape types. The relationship between behavior and emotion soundscapes (r = 0.22) further supports that social activities in public spaces tend to generate more emotional responses, especially in areas with cultural and historical significance.

The absence of memory-type soundscape across all neighborhoods suggests that social media data has limitations in capturing long-term acoustic associations with places. This gap indicates that a comprehensive soundscape assessment requires approaches that can access the temporal depth of the relationship between places and sounds. It also illustrates that social media analysis serves a complementary purpose in soundscape research rather than a primary method. Yet, these findings demonstrate that soundscape analysis based on social media can capture the sociocultural dimensions of urban acoustic experience. This shows how urban functions and cultural contexts create different soundscapes perceived as important by urban residents in their daily lives.

### 3.3. Urban soundscape patterns by clustering analysis

**Fig 9:**
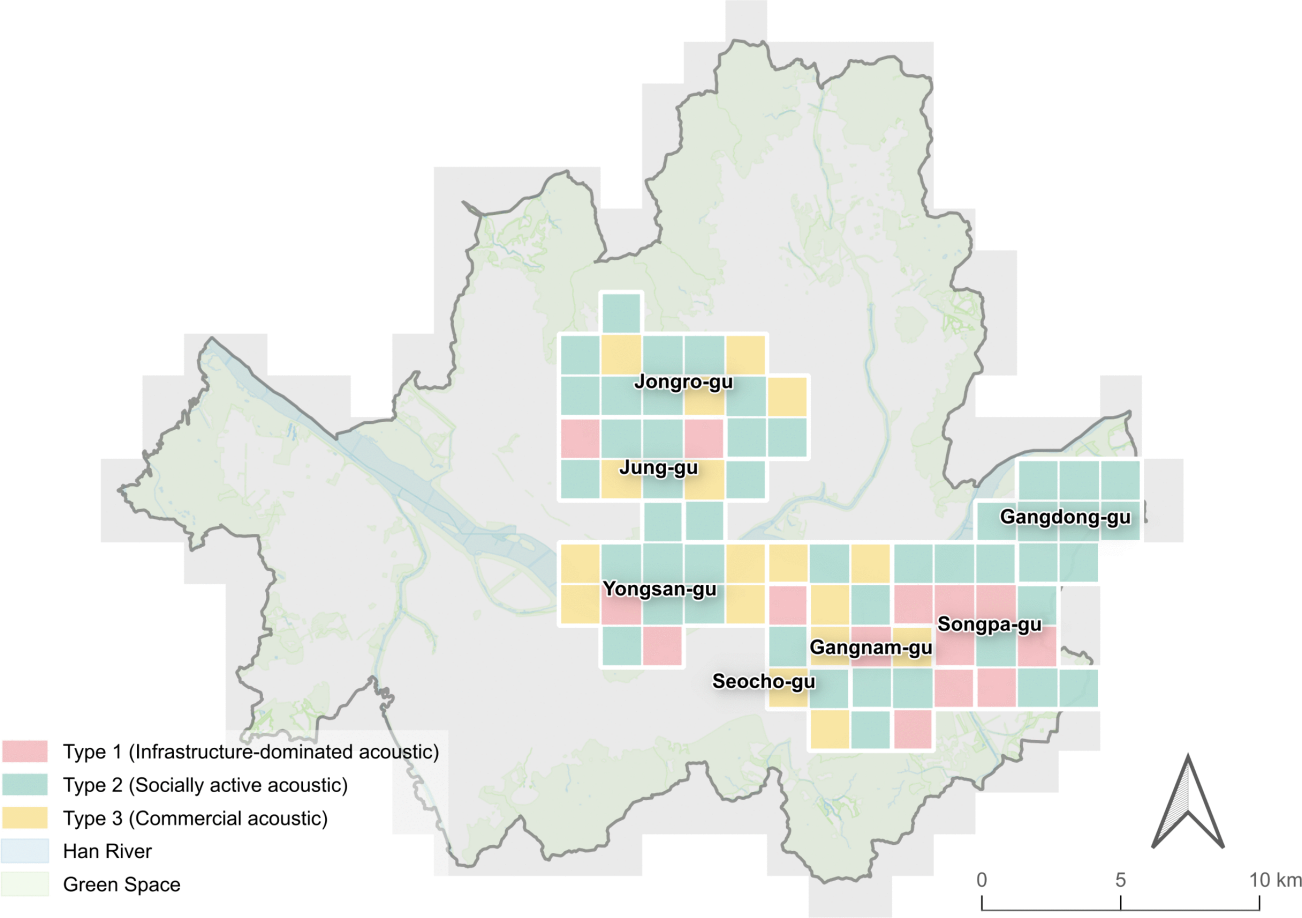
Urban soundscape patterns by district. This map shows 77 sub-regions classified into three distinct patterns (Type 1, Type 2, and Type 3) based on K-means clustering results. To compare the spatial distribution of soundscape patterns, the target area is represented as 1.5 × 1.5 ${𝐤𝐦}^{\text{2}}\;$ grid cell units; Source: Ministry of the Interior and Safety (http://www.juso.go.kr/externalLink/goUrl.do?menuId=DT05) and Seoul Metropolitan Government (https://data.seoul.go.kr/dataList/OA-21136/S/1/datasetView.do). These public works are used according to Korea Open Government License (KOGL).

**Fig 10:**
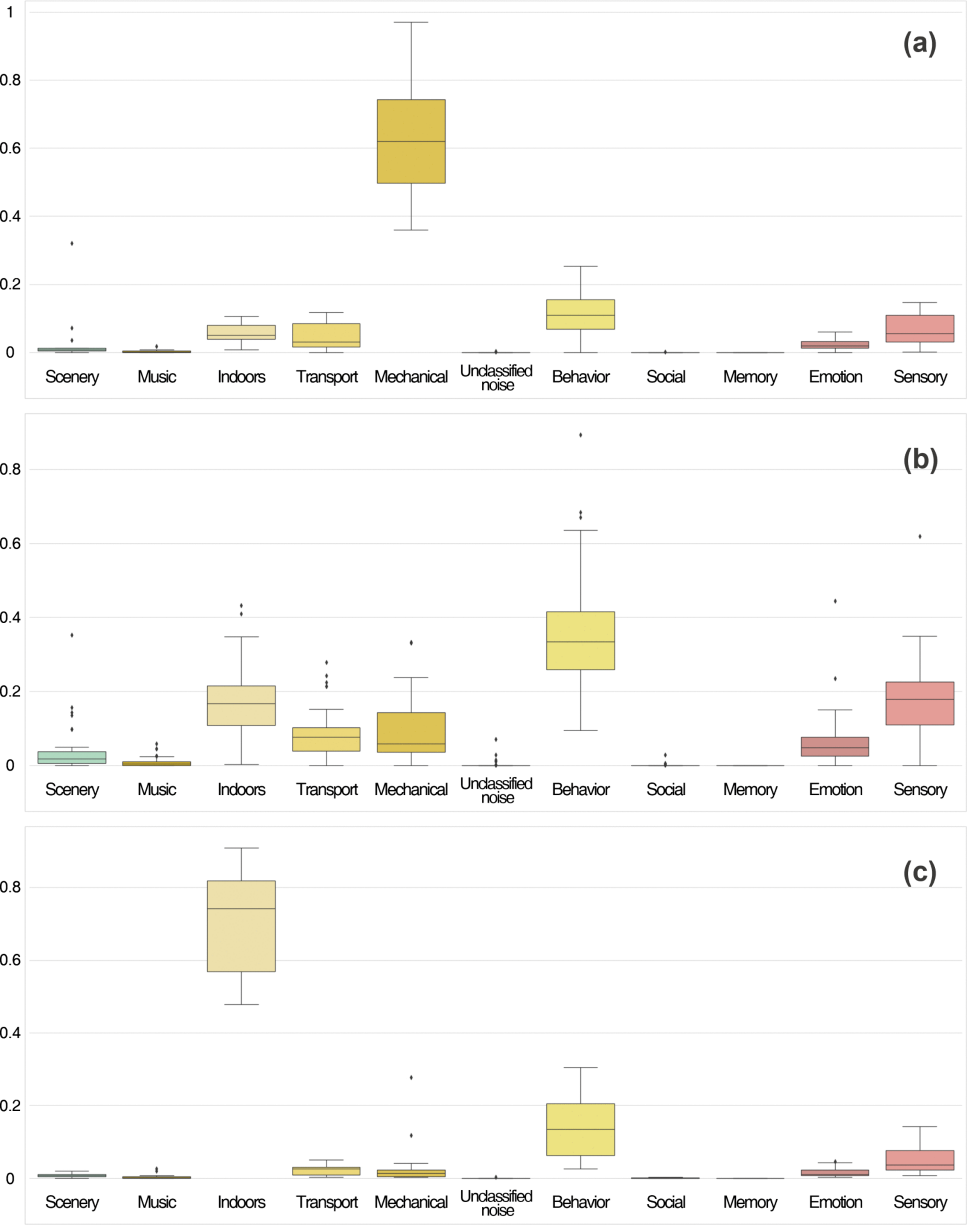
Distributions of the proportion of soundscape in each cluster. This box plot shows the mean proportion of soundscape categories for each cluster corresponding to the areas. **(a)** Type 1; **(b)** Type 2; **(c)** Type 3. X-axis: Soundscape types; Y-axis: Soundscape proportion.

Clustering analysis identified soundscape typologies in Seoul’s target areas, discovering how people perceive and experience urban acoustic environments. K-means clustering was applied to 77 dongs based on their soundscape category proportions, with the analysis revealing three distinct clusters (Type 1: n = 14, Type 2: n = 46, Type 3: n = 17). Despite urban soundscape perceptions being diverse and contextual, they were organized based on three key spatial-acoustic relationships that correspond to different urban functions. Figs 9 and 10 illustrate the soundscape patterns and the distribution of soundscape categories in each cluster.

ANOVA results confirmed significant differences between clusters for five soundscape categories (Table 4): indoors (F = 206.30, p < 0.001), mechanical (F = 148.76, p < 0.001), behavior (F = 26.23, p < 0.001), sensory (F = 15.52, p < 0.001), and transport (F = 8.28, p < 0.001). Post-hoc tests revealed distinct patterns: indoors category showed substantial differences among all three clusters (p < 0.05), demonstrating a spatial distinction. Mechanical category differed significantly between Type 1 and 2, and Type 1 and 3 (p < 0.001). Behavior and sensory categories primarily differentiated Type 2 from both Type 1 and 3 (p < 0.001), emphasizing Type 2’s unique profile. Transport showed significant difference only between Type 2 and 3. These results provide a useful framework for understanding soundscape patterns on a larger scale. The observed patterns also associate with relatively strong negative correlations found between indoor-mechanical (r = −0.46), mechanical-behavior (r = −0.45), and indoor-behavior (r = −0.39) soundscapes, indicating that these acoustic elements tend to occupy different urban spaces (Fig 8).

**Table 4:** The results of ANOVA and Tukey’s HSD.

Soundscape category	ANOVA		Tukey’s HSD		
	F-statistic	p-value	Group 1	Group 2	p-value
Scenery	1.5535	0.2183	1	2	0.9970
			1	3	0.4035
			2	3	0.2034
Music	1.9266	0.1529	1	2	0.2288
			1	3	0.9599
			2	3	0.3284
Indoors	206.3009	0.0000**	1	2	0.0012*
			1	3	0.0000**
			2	3	0.0000**
Transport	8.2813	0.0006**	1	2	0.088
			1	3	0.4319
			2	3	0.0006**
Mechanical	148.7573	0.0000**	1	2	0.0000**
			1	3	0.0000**
			2	3	0.1356
Noise	0.9502	0.3913	1	2	0.5622
			1	3	0.9994
			2	3	0.4881
Behavior	26.2313	0.0000**	1	2	0.0000**
			1	3	0.8017
			2	3	0.0000**
Social	0.5160	0.5990	1	2	0.6119
			1	3	0.9395
			2	3	0.8252
Memory	N/A^a^				
Emotion	4.9422	0.0097*	1	2	0.0788
			1	3	0.9543
			2	3	0.0213*
Sensory	15.5194	0.0000**	1	2	0.0004**
			1	3	0.9433
			2	3	0.0000**

*p < 0.05

**p < 0.001

a This category was not shown in all target areas.

#### 3.3.1. Type 1: Infrastructure-dominated acoustic zones *(High mechanical, low indoors).*

Type 1 areas, primarily found in Songpa-gu and the residential zones of Gangnam-gu, are defined by soundscapes dominated by mechanical activity. These areas showed the highest proportion of mechanical sounds (65.1%) and the lowest frequency of indoor acoustic elements (5.6%), reflecting the strong negative correlation between these two categories (r = −0.46). The strong presence of mechanical sounds and the low frequency of indoor acoustic elements suggest that construction and urban infrastructure shape the main auditory features of these spaces. The overall lower soundscape frequency in these areas indicates that people are less likely to share their acoustic experiences on social media, possibly because these environments are places where they pass through rather than engage with socially. Behavioral soundscapes comprised only 11.3% in these areas, consistent with the negative correlation between mechanical and behavior categories (r = −0.45), while sensory experiences were also limited (6.8%). These findings highlight the importance of implementing acoustic management in highly developed areas to support urban functionality and improve residents’ quality of life.

#### 3.3.2. Type 2: Socially active acoustic environments *(High behavior).*

Type 2, which includes the majority of districts (59.7% of all target areas), reflects Seoul’s main acoustic environment dominated by human activity sounds. These areas demonstrated the highest proportions of behavior-related soundscapes (35.9%), followed by sensory experiences (17.6%), significantly higher than other clusters (p < 0.001). These balanced variety of sounds, with consistently higher levels in all sound categories compared to other cluster types, points to places where complex and diverse soundscapes are shaped by everyday human activity. The moderate levels of indoor sounds (17.2%) combined with low mechanical presence (9.7%) create an acoustic environment that can support social interaction without the dominance of either commercial indoor spaces or infrastructure noise. The frequent use of behavioral terms (e.g., events, voices, movement, adults, and children) combined with the statistical prominence of emotion-related sounds (6.2%) suggests these areas function as key social and cultural centers. This pattern illustrates that urban soundscapes have potential for social engagement with their auditory features.

#### 3.3.3. Type 3: Commercial acoustic spaces *(High indoors, low mechanical).*

Type 3 areas focus on indoor soundscapes with limited mechanical noise, reflecting controlled environments adapted for social and commercial use. The large dominance of indoor sounds (70.9%) contrasted sharply with minimal mechanical presence (3.5%), representing the strongest differentiation among all clusters and confirming their negative correlation. The frequent mention of cafés, restaurants, and stores highlights places where indoor acoustic settings support both interaction and economic activity. Especially, these commercial spaces showed the lowest sensory soundscape proportions (5.8%) compared to Type 2’s 17.6%, suggesting that controlled commercial environments may limit diverse sensory experiences despite their social functions. These areas show high soundscape frequencies, indicating strong engagement on social media and revealing that their acoustic environments encourage the sharing of experiences. The moderate behavior levels (14.5%) combined with high indoor proportions reflect the negative correlation (r = −0.39) between these categories, confirming that users focus more on indoor commercial experiences rather than outdoor social activities in these spaces.

## 4. Discussion

### 4.1. Interpretation of urban soundscape patterns

This study identified three distinct urban soundscape patterns through k-means clustering analysis of Seoul’s central and southeastern districts. Infrastructure-dominated zones (Type 1) showed high mechanical sounds (65.1%) with minimal indoor activity (5.6%), mainly found in Songpa-gu and residential areas of Gangnam-gu. These spaces represent areas where mechanical sounds dominate human activities, with minimal behavioral soundscapes (11.3%) and limited sensory experiences (6.8%). This pattern indicates that infrastructure noise may reduce social engagement and quality of urban life [5,6], with social media data highlighting the absence of positive acoustic experiences rather than the presence of negative ones.

Socially active environments (Type 2) comprised the majority (59.7%) of analyzed spaces, exhibiting high behavioral (35.9%) and sensory (17.6%) soundscapes with moderate indoor sounds (17.2%) and low mechanical presence (9.7%). This dominance displays that socially active acoustic environments constitute Seoul’s primary soundscape experience. The high proportions of behavioral and sensory soundscapes support the concept of urban vibrancy proposed by Aletta & Kang [58], where human presence and activities serve as positive acoustic indicators instead of noise sources. The positive correlation between behavior and emotion soundscapes (r = 0.22) further suggests that spaces supporting social activities tend to generate emotional responses, particularly in areas with cultural significance. This finding corresponds to Gatti & Procentese [59], who presented that social media engagement with local places strengthens sense of place and community connections.

Commercial spaces (Type 3) were characterized by dominant indoor sounds (70.9%) with minimal mechanical presence (3.5%), representing the strongest differentiation among clusters. While this dominance implies acoustic insulation from urban noise, these spaces showed the lowest sensory soundscape proportions (5.8%) compared to Type 2 (17.6%). While controlled commercial environments support social and economic functions, they may limit diversity of sensory experiences. Aletta et al. [60] demonstrated that acoustic interventions in transit spaces could extend dwell time without architectural changes. Our findings reveal similar potential in commercial spaces, but the low level of sensory engagement indicates that acoustic comfort alone may not create memorable urban experiences.

The strong negative correlations between indoor-mechanical (r = −0.46), mechanical-behavior (r = −0.45), and indoor-behavior (r = −0.39) soundscapes confirm these acoustic elements occupy spatially separate zones. This suggests that urban acoustic zones emerge from functional specialization rather than the result of deliberate acoustic planning, highlighting opportunities for more intentional soundscape design [58].

Central districts with rich historical heritage (Jongro-gu and Jung-gu) demonstrated significantly higher proportions of meaning-related soundscapes compared to southeastern areas (Mann-Whitney U test, p = 0.004). Specifically, emotion-related soundscapes were nearly twice as prevalent in central areas (5.8% vs 3.3%), while sensory-related soundscapes also presented higher prevalence (16.2% vs 10.2%). This pattern is consistent with the concept of acoustic heritage emphasized by Maffei et al. [61], where soundscapes constitute an integral component of cultural identity. The prominence of sensory and emotional descriptions in these areas shows that social media users document an experience that covers physical sounds and their cultural and personal significance. However, this interpretation should consider that heritage sites typically attract more tourists and visitors who may be more inclined to share experiential content on social media.

### 4.2. Methodological contributions and limitations

These interpretations require consideration of methodological constraints and cultural context. Most critically, reliance on Twitter as the sole data source introduces substantial limitations. Twitter’s declining user base and demographic bias toward younger, urban populations may not represent the full spectrum of urban soundscape experiences [34,35]. The platform’s text-based nature and character limitations may also discourage detailed acoustic descriptions, potentially explaining the low frequency of natural soundscapes even in areas with parks and green spaces.

The data collection period (October-November 2020) coincided with the COVID-19 pandemic, introducing a temporal specificity that limits generalizability. Lee & Jeong [62] demonstrated significant changes in urban noise perception during lockdowns, with outdoor environments becoming quieter while neighbor noise gained prominence. The high proportion of indoor soundscapes in our data may reflect pandemic-related behavioral changes than typical urban acoustic patterns. Furthermore, restrictions on social gatherings during this period have likely changed the acoustic characteristics of public spaces, potentially not reflecting the vibrancy of social activities in Type 2 areas.

Cultural context presents additional consideration. Aletta et al. [63] demonstrated significant differences in soundscape perception between European and Chinese populations, where Europeans associated vibrancy with human sounds while Chinese participants linked it to natural sounds. Korean urban culture, with its unique combination of traditional and modern elements, may produce distinct soundscape preferences not captured by Western contexts. The high proportion of indoor soundscapes in commercial areas might reflect Korean café culture and the social importance of indoor gathering spaces, instead of universal patterns. Future applications of Canter’s place model [25] should consider such cultural variations, potentially requiring regional adaptations of the taxonomy to capture culturally specific acoustic meanings.

The absence of memory-type soundscapes among all neighborhoods and the limited presence of natural sounds confirms fundamental limitations of social media-based soundscape research. Unlike standardized soundwalk methodologies [29,32] or the comprehensive PHS (Physical, Historical, Social) approach proposed by Maffei et al. [61], social media data reflects selective sharing behaviors. Users appear to prioritize documenting socially engaged experiences over ambient environmental sounds. While this selectivity provides insights into which sounds people find meaningful enough to share, it cannot replace systematic acoustic assessment methods that capture the full spectrum of urban soundscapes.

Despite these limitations, this study contributes methodologically by demonstrating scalable soundscape assessment beyond traditional point-based measurement. Previous studies struggled with limited geotagged data, often restricting analysis to small areas or specific locations [17,35,38]. Our POI-based georeferencing approach, linking texts to 156 POIs across seven districts, expanded spatial coverage while maintaining location specificity. The significant correlation between POI density and soundscape frequency (Spearman ρ = 0.43, p < 0.001) validates this method’s effectiveness in capturing localized acoustic perceptions. This statistical relationship serves as an indirect validation of our core assumption that not all posts may reflect direct experiences. While this provides confidence in our large-scale approach, further research employing manual validation or sampling would be beneficial to directly prove the context of individual posts. This scalability of a POI-based approach supports the need for area-wide soundscape characterization in urban planning, though it sacrifices the acoustic precision of ISO 12913 standard methods.

Applying Canter’s place model [25] to soundscape taxonomy provides another contribution, integrating physical, behavioral, and semantic dimensions in a unified framework. While previous classifications focused on sound sources [10,16–18] or perceptual dimensions such as pleasantness-eventfulness models [19,20], our approach captures the contextual nature of soundscape perception. This framework particularly complements the holistic approach advocated by Aletta et al. [64], which emphasizes that soundscape quality depends on acoustic factors and visual, spatial, and social contexts. The relationships between visual quality and soundscape satisfaction shown in tourist sites support our finding that meaning-related soundscapes concentrate in visually distinctive heritage areas [64].

The clustering analysis revealing three distinct patterns provides a data-driven typology that could serve as a potential screening tool for urban planners. Rather than replacing detailed acoustic measurements, this approach could identify areas requiring focused investigation using standardized methods such as soundwalks or the Management Plan for heritage sites [61]. For instance, Type 1 areas with high mechanical soundscapes would benefit from objective noise measurements and psychoacoustic analysis to quantify the actual impact on residents’ wellbeing [40]. Similarly, the vibrancy characteristics identified in Type 2 areas should be validated through on-site soundwalks following ISO/TS 12913−2 protocols, combining binaural recordings with perceptual assessments to confirm whether social media-derived patterns align with in-situ experiences [26,63,65]. The discrepancy between high indoor soundscape proportions in Type 3 commercial areas and low sensory engagement particularly warrants acoustic simulation studies to understand how architectural and acoustic design influences perceptual diversity [58]. Acoustic zones emerge from functional specialization without deliberate planning, suggesting opportunities for implementing comprehensive databases documenting acoustic characteristics, sound sources, and sound marks in urban districts, extending heritage management to broader urban contexts [61].

## 5. Conclusions

This study examined how urban soundscapes are perceived using social media data, demonstrating that these perceptions follow distinct patterns closely associated with the spatial functions of the city. The results identified three primary acoustic environments within Seoul: infrastructure-dominated, socially active, and commercial zones, which reveals how urban identity is reflected in the collective acoustic experience. Methodologically, this study contributes a scalable POI-based approach that expands the spatial scope of soundscape analysis. Additionally, the application of Canter’s place model provides a more comprehensive framework for interpreting the human experience of acoustic environments. For urban planners and policymakers, these findings suggest that social media analysis can serve as a valuable complementary tool for potential site assessments. This approach presents insights into citizens’ perceptions that can enhance discussions on urban acoustic heritage. Future research should validate these findings by integrating multi-platform social media data with objective acoustic measurements, including ISO/TS 12913−2 methods [26]. Post-pandemic analysis is also necessary to distinguish long-term soundscape patterns from temporary phenomena and develop a more robust understanding of the urban acoustic environments.

## Supporting information

S1 Appendix Table A & table B. (ZIP)
